# Annotations

**Published:** 1898-07-23

**Authors:** 


### Annotations.

### Women and Dangerous Trades.

A meeting which was recently held in the Jerusalem Chamber, Westminster Abbey, ended by resolving to appoint a deputation to wait upon the Home Secretary, praying for the total legislative prohibition of the use of poisonous phosphorus, and of the importation of matches made by poisonous phosphorus. The conjunction of these two items is worthy of note, and those who in the presence of great evils and abuses would, as is so easy, call down laws from heaven to put things right instead of taking the trouble to put them right themselves, will do well to observe how, by its wording, this resolution recognises the fact that the legislative prohibition of the use of poisonous phosphorus in the making of matches in England would merely have the effect of throwing English match-makers out of work and handing the trade over to the foreigner unless coupled with legislative protection for other forms of matches. Common sense says that if the laws which have been passed for the protection of women and children have been broken, those who have done this thing should be hunted down and made examples of, and that all good citizens should take care that such law-breaking is not repeated.

### Breaches of the Factory Acts.

At the meeting above referred to another resolution was proposed by the Baroness Burdett-Coutts and seconded by the Duchess of Sutherland, which was far more to the point. It was that a committee be formed to "watch over the interests of women engaged in dangerous trades." The proposer of this resolution very justly said that "no proper remedy would be provided until they had something in addition to Factory Acts and inspectors. There might," she added, "be means devised of obtaining information through another separate body." This goes to the root of the difficulty. Those who know the provisions of the law admit that, if properly put in force, it ought to afford protection. It is the information by which the law is set in action that is wanted. It is a sad thing, and a terrible thing, that the competition for employment should be so keen that workpeople employed in dangerous trades should hesitate and hold back from giving information regarding breaches of the regulations imposed by law for their own safety; but so it is. What is wanted is that people of influence and power should make themselves the friends of these girls, become their confidants, and discover what is the inner life of these factories, which seems so closed a book to the ordinary inspector. As the Duchess of Sutherland said, they "needed something more than subscription lists and soup kitchens; they needed the intelligent, determined help of men and women who were willing to give their lives to stamp down cruelty and falsehood." The price of public health, as of public liberty, is eternal vigilance. Not in a multitude of laws, but in a multitude of watchers safety lies. At a meeting of the Industrial Law Committee which was held last Tuesday, under the presidency of the Bishop of London, another step was taken which may have an important bearing on the same question. It was decided to establish an indemnity fund for the protection of women and children who suffer dismissal in consequence of giving evidence which is required by law. This will, no doubt greatly strengthen the position of those who are engaged in dangerous trades, and it is worthy of note that Mr. Asquith in seconding the resolution said that it had been his lot for some years to superintend the administration of the laws which had been passed for the protection of the working members of our industrial community, and that although inspection was well and wisely done, it was in many cases rendered nugatory by the impossibility of obtaining sufficient proof of the breaches of the law that were suspected. Prosecution, he said, could not be undertaken on suspicions, and convictions could not be obtained on moral certitude. As to the suggestion that such action as was proposed might tend to embitter the employers against the employed, he pointed out that the law was already observed spontaneously by every good employer, because it was in consonance with the dictates of justice and of common sense.

### Hypnotism.

Dr. Kingsbury is much to be congratulated on the result of the trial in regard to the validity of the will of Mrs. Howard, according to which he is understood to benefit to the extent of somewhere between £30,000 and £40,000. Nevertheless, we cannot but feel that the moral of the whole case must not be taken as a hint to the profession to go and do likewise, for there is much in it to show that the practice of hypnotism, even as a mere therapeutic measure, is surroundedby many dangers which no practitioner should lightly run. The jury could hardly have given any other verdict than the one which they returned unless they had utterly disbelieved in Dr. Kingsbury. The judge pointed out that the question turned upon whether or not the doctor had been guilty of "undue influence" in obtaining the residuary clause, and he also said that "he could not help thinking that this case almost entirely depended upon the view they took of Dr. Kingsbury himself, whether they really trusted him as a witness, or whether they did not"; and under such circumstances, gratified as all Dr. Kingsbury's friends must feel at the verdict which was ultimately arrived at, it is impossible to shut one's eyes to the risks he ran, as shown by the long deliberations of the jury before a verdict was arrived at; and one cannot but feel that the suspicion in which the practice of hypnotism is generally held had much to do with their hesitancy in the matter. The fact is that at the present time, although hypnotism is well recognized by the profession as a reality, and as a power capable of doing good in some cases, at any rate for the time, it is also recognised as a proceeding which is so capable of abuse that, pending more complete knowledge, it would be wise to restrict its use to cases in which it may be employed in a purely scientific manner, and in regard to which no ulterior motives can even be suggested. So far as the one in question is concerned we do not believe that hypnotism had anything whatever to do with the matter before the Court, but we do think that the natural and proper suspicion in which hypnotism is held by the public had much to do with making it possible for the defendants to get up the case they did, and we would urge practitioners so to limit their hypnotic experiments that even their enemy shall be unable to suggest motives which are not good.

